# *Cercopithifilaria* spp. of dogs: little known but prevalent filarioids beneath the skin

**DOI:** 10.1186/s13071-023-06007-5

**Published:** 2023-10-25

**Authors:** Marcos Antonio Bezerra-Santos, Filipe Dantas-Torres, Rafael Antonio Nascimento Ramos, Emanuele Brianti, Domenico Otranto

**Affiliations:** 1 Department of Veterinary Medicine, University of Bari, Valenzano, Bari, Italy; 2 Aggeu Magalhães Institute, Fundação Oswaldo Cruz (Fiocruz), Recife, Brazil; 3 Federal University of the Agreste of Pernambuco (UFAPE), Garanhuns, Brazil; 4 Department of Veterinary Science, University of Messina, Messina, Italy; 5 Department of Pathobiology, Faculty of Veterinary Science, Bu-Ali Sina University, Hamedan, Iran

**Keywords:** Subcutaneous filarioids, *Cercopithifilaria* spp., Nematodes, Dogs

## Abstract

Filarioids of the genus *Cercopithifilaria* are little studied, yet widespread parasites, that are relatively unique in being one of the very few nematodes transmitted by hard ticks. These filarioids live in the subcutis while microfilariae are found in the dermis. Definitive hosts include domestic dogs as well as a wide range of vertebrates, such as ruminants, non-human primates, murids, marsupials, porcupines, viverrids, bears and lagomorphs. The genus *Cercopithifilaria* contains three taxa (i.e. *C. bainae*, *C. grassii* and a yet undescribed species, namely *Cercopithifilaria* sp. II) that are known to infect dogs worldwide, with their occurrence overlapping the distribution of the main tick vector, *Rhipicephalus sanguineus* sensu lato. In recent decades, more attention has focused on these filarioids since they have been associated with clinical signs of infection, such as dermatitis, chronic polyarthritis and cutaneous cysts, and possibly with facilitating infections caused by other tick-borne pathogens. Nevertheless, these parasites remain largely underdiagnosed in clinical practice due to the lack of awareness of veterinary practitioners and to major obstacles to their diagnosis. In this review, we have assessed currently available data on *Cercopithifilaria* spp. infecting dogs worldwide and discussed the biological, clinical and epidemiological aspects of these filarioids, with the overall aim to gain a better understanding of their potential role in skin diseases.

**Graphical abstract:**

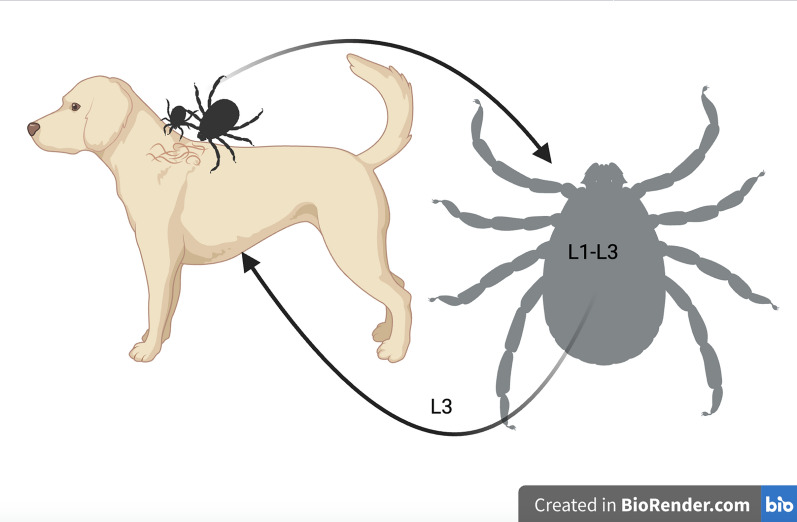

## Background

Canine filarioids are a group of vector-borne nematodes infecting dogs and other animal species worldwide, with the filarial worm *Dirofilaria immitis* being considered one of the most important mosquito-borne parasites due to the cardiopulmonary disease it may cause [1, 2]. Conversely, *Dirofilaria repens*, which localizes in subcutaneous tissues, causes a less relevant clinical form in dogs but is the major cause of zoonotic infection in humans [3]. Other less known filarioids that infect dogs, such as *Acanthocheilonema* spp., *Brugia* spp. and *Cercopithifilaria* spp., affect the subcutaneous tissues, muscular fasciae, body cavity, retropharyngeal and axillary lymphatics of carnivores and, in the case of *Onchocerca lupi*, also ocular tissues [1, 4, 5]. For most of the above-mentioned filarioids, information on the life history as well as the actual impact on human and veterinary medicine is meager.

Differently from the majority of the filarioids infecting dogs, whose microfilariae (mfs) circulate in the blood stream, *Cercopithifilaria* spp. and *O. lupi* share a unique characteristic in that they have dermal mfs. The zoonotic potential of *O. lupi* [6] has attracted the interest of the scientific community [5], with studies published over the last 20 years on many clinical aspects of the infection caused by this filarioid in both dogs [7] and cats from North America [8] and Europe [9, 10]. Nonetheless, the vector of this onchocercid is still unknown although *O. lupi* genomic DNA was molecularly detected in black flies (Simuliidae) [11], *Culex* spp. mosquitoes (Culicidae) [12] and biting midges (Ceratopogonidae) [13]. Conversely, there is a lack of awareness by veterinary practitioners about *Cercopithifilaria* spp., mostly due to its mild clinical significance and difficulties in diagnosing infections [14, 15]. Such difficulties are due to the presence of adults in the subcutis and mfs beneath dermal tissues [14, 16], and detection requires a rather invasive procedure [14]. To date, three species of *Cercopithifilaria* are known to infect dogs, with *C. bainae* being the most frequently detected, followed by *Cercopithifilaria grassii* and *Cercopithifilaria* sp. II sensu Otranto et al. [1] (from now on, referred to as “*Cercopithifilaria* sp. II”) [17]. These subcutaneous filarioids are transmitted by brown dog ticks (*Rhipicephalus sanguineus* sensu lato) (*R. sanguineus* s.l.), which are commonly found infesting dogs in tropical, subtropical and some temperate regions worldwide [18]. Many studies have demonstrated that the distribution of these filarioids overlaps that of *R. sanguineus* s.l., with reports in ticks and dogs from Africa [19], Asia [20], Middle East [21], Europe [15, 17, 19, 22–27], North America [28, 29] and South America [30–33] (Table 1).

**Table 1:** Molecular data on *Cercopithifilaria* spp. reported worldwide

Sample	*Cercopithifilaria* species	Geographic region	Gene	Genbank accession number	References
Ticks (*R.s.*)	*C.b*	Australia	*Cox*1	KJ460110	[19]
Ticks (*R.s.*)	*C.b*	Greece	*Cox*1	MT365663	[27]
Dog skin	*C.b*	Greece	*Cox*1	MT365664	[27]
Dog skin	*C.b*	Portugal	*Cox*1	99% identical with JF461457	[22]
Dog skin	*C.b*	Portugal	12S RNA	99% identical with JF461461	[22]
Dog skin	*C.b*	Romania	*Cox*1	100% identical with JF461457	[23]
Ticks (*D.r.*)	*C.b*	Romania	*Cox*1	MF479726	[68]
Dog skin	*C.b*	Italy	*Cox*1	KF270686	[26]
Dog skin	*C.b*	Italy	12S RNA	KF381408	[26]
Ticks (*R.s.*)	*C.b*	China	*Cox*1	ON176668	[20]
Ticks (*R.s.*)	*C.b*	India	*Cox*1	ON176670	[20]
Ticks (*R.h.*)	*C.b*	India	*Cox*1	ON176671	[20]
Ticks (*R.s.*)	*C.b*	Indonesia	*Cox*1	ON176663	[20]
Ticks (*R.s.*)	*C.b*	The Philippines	*Cox*1	ON176665	[20]
Ticks (*R.s.*)	*C.b*	Taiwan	*Cox*1	ON176669	[20]
Ticks (*R.s.*)	*C.b*	Vietnam	*Cox*1	ON176667	[20]
Dog skin	*C.b*	USA	*Cox*1	MH390716	[28]
Ticks (*R.s.*)	*C.b*	USA	*Cox*1	95–100% identical with MF479726, JQ305156 and JQ305157	[29]
Dog skin	*C.b*	Costa Rica	*Cox*1	100% identical with JF461457	[30]
Dog skin	*C.b*	Costa Rica	12S RNA	100% identical with JF461461	[30]
Dog skin	*C.b*	Brazil	12S RNA	KX156956	[31]
Ticks (*R.s.*)	*C.b*	Brazil	12S RNA	KY083056	[32]
Ticks (*R.s.*)	*C.g*	Pakistan	*Cox*1	KJ460111	[19]
Ticks (*R.s.*)	*C.g*	The Philippines	*Cox*1	ON176672	[20]
Ticks (*R.s.*)	*C.g*	India	*Cox*1	ON176673	[20]
Dog skin	*C.g*	Italy	*Cox*1	JQ837810	[17]
Dog skin	*C.g*	Portugal	*Cox*1	99% identical with JQ837810	[22]
Dog skin	*C.g*	Portugal	12S RNA	99% identical with JQ837812	[22]
Dog skin	*C.*spII	Italy	*Cox*1	JQ837809	[17]
Dog skin	*C.*spII	Portugal	*Cox*1	99% identical with JQ837809	[22]

*R.s. Rhipicephalus sanguineus* Sensu lato, *R.h. Rhipicephalus haemaphysaloides*, *D.r. Dermacentor reticulatus*, *C.b. Cercopithifilaria bainae*, *C.g. Cercopithifilaria grassii*, *C.*spII *Cercopithifilaria* sp. II

In this article we review the current data available for *Cercopithifilaria* spp. affecting dogs worldwide, discussing the biological, clinical and epidemiological aspects of these subcutaneous filarioids, with the overall aim to gain a better understanding of the role these parasites may play in dermal pathologies.

## The genus* Cercopithifilaria*

The genus *Cercopithifilaria* comprises 28 species that have been described in several vertebrate hosts, such as domestic and wild ruminants, domestic dogs, non-human primates, murids, marsupials, porcupines, viverrids, bears and lagomorphs (Table 2). These subcutaneous filarioids were previously classified as a subgenus within the genus *Dipetalonema* [34] and later elevated to the genus *Cercopithifilaria* by Bain et al. in 1982 [35]. This taxonomical reconsideration was based on the morphological differences between the two genera, with *Cercopithifilaria* spp. presenting a slender esophagus, very small buccal capsule, a straight spicule of stubby shape, without distinct handle and caudal papillae often in reduced number and gathered near the cloaca [35].

**Table 2:** *Cercopithifilaria* spp. described to date, with their hosts and tick vectors

Species	Hosts	Tick vector	References
*Cercopithifilaria bainae*	Dogs	*Rhipicephalus sanguineus* sensu lato	[14, 57]
*Cercopithifilaria grassi*	Dogs	*Rhipicephalus sanguineus* sensu lato	[50]
*Cercopithifilaria* sp. II	Dogs	*Rhipicephalus sanguineus* sensu lato	[17]
*Cercopithifilaria longa*	Ruminants	Unknown	[36]
*Cercopithifilaria ruandae*	Ruminants	Unknown	[36]
*Cercopithifilaria dermicola*	Ruminants	Unknown	[38]
*Cercopithifilaria faini*	Ruminants	Unknown	[36]
*Cercopithifilaria bulboidea*	Ruminants	Unknown	[36]
*Cercopithifilaria crassa*	Ruminants	Unknown	[36]
*Cercopithifilaria multicauda*	Ruminants	Unknown	[37]
*Cercopithifilaria minuta*	Ruminants	Unknown	[36]
*Cercopithifilaria tumidicervicata*	Ruminants	Unknown	[36]
*Cercopithifilaria rugosicauda*	Ruminants	*Ixodes ricinus*	[67, 73]
*Cercopithifilaria shohoi*	Ruminants	Unknown	[74]
*Cercopithifilaria cephalophi*	Ruminants	Unknown	[35]
*Cercopithifilaria degraaffi*	Non-human primates	Unknown	[42]
*Cercopithifilaria eberhardi*	Non-human primates	Unknown	[42]
*Cercopithifilaria kenyensis*	Non-human primates	Unknown	[34, 42]
*Cercopithifilaria narokensis*	Non-human primates	Unknown	[42]
*Cercopithifilaria verveti*	Non-human primates	Unknown	[43]
*Cercopithifilaria corneti*	Viverrids	Unknown	[47]
*Cercopithifilaria johnstoni*	Murids and marsupials	*Ixodes trichosuri*	[45]
*Cercopithifilaria didelphis*	Marsupials	Unknown	[35]
*Cercopithifilaria pearsoni*	Marsupials	Unknown	[44]
*Cercopithifilaria gabonensis*	Porcupines	Unknown	[35]
*Cercopithifilaria roussilhoni*	Porcupines	*Rhipicephalus sanguineus* sensu lato	[46]
*Cercopithifilaria japonica*	Bears	Unknown	[48]
*Cercopithifilaria leporinus*	Lagomorphs	Unknown	[49]

Based on current knowledge, ruminants are the vertebrate hosts that harbor almost half of the species described in the genus *Cercopithifilaria* (Table 2), although data on the clinical aspects are not available for any of these species [36]. For example, the Japanese serow (*Capricornis crispus*) may be parasitized by five different *Cercopithifilaria* species (i.e. *C. shohoi*, *C. multicauda*, *C. minuta*, *C. tumidicervicata* and *C. bulboidea*), frequently simultaneously [37]. In addition, two other species (i.e. *C. longa* and *C. crassa*) have been described in sika deer (*Cervus nippon nippon*) from Japan, bringing the number of *Cercopithifilaria* species occurring in ruminants from Asia to a total of seven [36]. Another four species have been described in ruminants from Africa (i.e. *C. ruandae* and *C. dermicola* in cattle, *C. faini* in *Cephalophus nigrifrons* and *C. cephalophi* in *Cephalophus dorsalis* antelopes) [35, 36, 38]. Finally, a single species has been described in Europe (i.e. *C. rugosicauda*), parasitizing the roe deer *Capreolus capreolus* [39]. The latter is vectored by *Ixodes ricinus* ticks; this is the only *Cercopithifilaria* sp. of ruminants whose vector is currently known [40, 41].

In non-human primates, five *Cercopithifilaria* species have been described, with *C. kenyensis*, *C. narokensis* and *C. eberhardi* infecting baboons (*Papio* spp.), and *C. verveti* infecting the vervet monkey (*Cercopithecus aethiops*) from Africa [35, 42, 43]. In addition, three *Cercopithifilaria* species have been described in marsupials from Australia (i.e. *C. johnstoni* and *C. pearsoni* [44, 45]) and Colombia (i.e. *C. didelphis* [35]), two (i.e., *C. gabonensis* and *C. roussilhoni* [35, 46]) in porcupines from Africa and other three species in viverrids (i.e. *C. corneti* [47]), bears (i.e. *C. japonica* [48]) and lagomorphs (i.e. *C. leporinus* [49]). The *Cercopithifilaria* spp. infecting dogs will be discussed in the following sections.

## *Cercopithifilaria* spp. in dogs: the end of almost a century of silence

The first *Cercopithifilaria* species known to infect dogs, namely *C. grassii* (= *Filaria grassii* Noè, 1907) is characterized by typical mfs, which were defined “gigantesche” (from the Italian word for giant) and with a “gland shape head” by Noè [50, 51]. These early studies provided a detailed description of adults and immature stages of *C. grassi*, along with information on the vector role of *R. sanguineus* s.l.

*Cercopithifilaria grassii* remained completely ignored until the early 1980s, when larval stages were reported in *R. sanguineus* s.l. ticks from Switzerland [52] and in dogs from Brazil [53]. In subsequent years, this species was detected in ticks from northern Italy [54], and another canine filarioid was described and named as *Cercopithifilaria bainae* in Brazil [55]. Following these early reports, these nematodes remained undiagnosed in dogs until 2011, when a study was conducted in Sicily [56]. The study initially attempted to demonstrate the role of *R. sanguineus* s.l. ticks as vectors of *Acanthocheilonema reconditum* by experimentally infesting two highly microfilaremic dogs with *R. sanguineus* s.l. nymphs [57]. Indeed, although it is well established that *A. reconditum* is vectored by fleas and lice [58], few reports hinted at the role of ticks [59, 60]. Therefore, the observation of developing larvae of filarioids only in ticks fed on one of the two dogs with *A. reconditum* circulating mfs suggested that the one dog (named Margherita) was infected by another filarioid. This was a serendipity finding which allowed researchers to morphologically and molecularly identify the dermal mfs detected in the dog as *Cercopithifilaria* sp. I sensu Otranto et al. [56]. The same parasite was later redescribed as *C. bainae*, along with a detailed morphological and molecular characterization [61], which was missing in the original description [55]. Finally, mfs of a third putative species, named *Cercopithifilaria* sp. II, were morphologically and molecularly detected in dogs from the Mediterranean area [61]. However, in the latter case, the lack of available adult specimens impeded a formal species description and therefore its taxonomical position remains undefined.

To date, information on the biology, epidemiology and clinical aspects of these filarioids in dogs is scarce and mostly available for *C. bainae* due to its wide distribution when compared to *C. grassii* and *Cercopithifilaria* sp. II. Indeed, the real burden caused by these parasites on infected dogs is unknown and deserves attention by the scientific community.

## Morphology of adult *C. bainae* and *C. grassii*

**Fig. 1:**
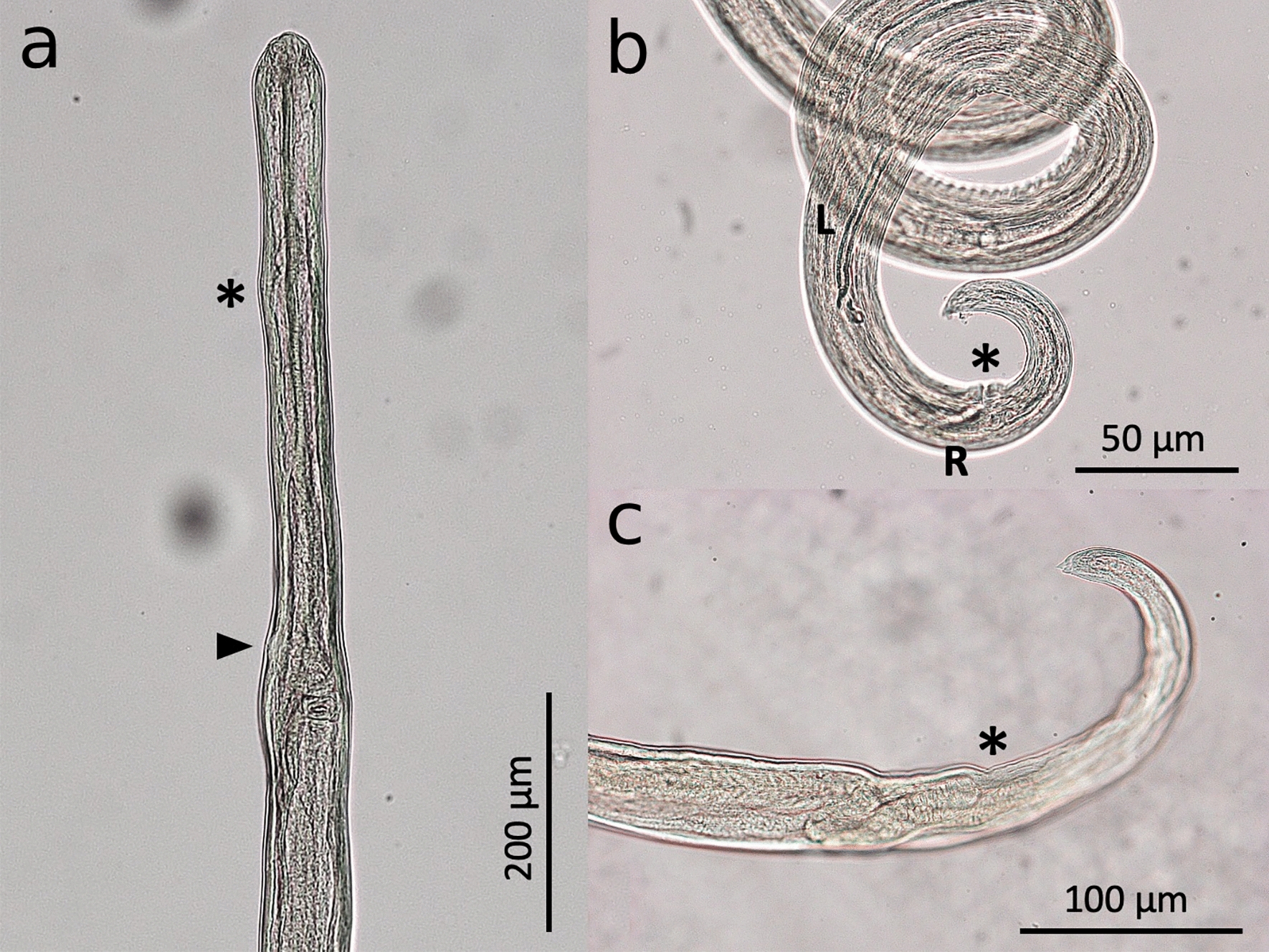
Adults of *Cercopithifilaria bainae*. **a** Cephalic region of female, lateral view; note the level of nervous ring (asterisk) and vagina (arrow). **b** Caudal region of male, lateral view; note that the left spicule (L) is longer than the right one ®) at the anus level (asterisk). **c** Caudal region of female, lateral view; note the anus level (asterisk)

*Cercopithifilaria bainae* and *C. grassii* adults present important morphological and morphometrical characters that allow their differentiation into distinct species. Briefly, *C. bainae* adults are slender and delicate, with a conical anterior end, having four external labial and four cephalic submedian papillae and one pair of amphids (Fig. 1a). The oral opening of this species is small and round, presenting a flattened ring-like buccal capsule [61]. The esophagus contains numerous glandular cells in the posterior part, with a nerve ring at the level of anterior and middle third portions, and the lumen is flattened dorsoventrally (Fig. 1a). This species also presents a thin and smooth cuticle, and the tail is curved ventrally with two short and one longer dorsal conical cuticular processes known as lappets at the tail tip, and phasmids at the base of lateral lappets [61]. Males of *C. bainae* present a single ventral median precloacal papilla and five to six pairs of caudal papillae. The right spicule is shorter and has a conspicuous dorsal heel; the left spicule presents a hook at the distal end (Fig. 1b). Females have didelphic opistodelphic reproductive system, with a slit-like vulva, a muscular vagina and an ovijector directed posteriorly with the presence of circular muscular walls. The tail is long, slender and bent ventrally (Fig. 1c) [61].

Adults of *C. grassii* have been described by Noé [50] as presenting an anterior extremity that is larger than the posterior one, with four cephalic papillae. The cephalic end is distinguished from the remaining body by a retrocephalic narrowing (only in females). Females also present a cylindric body with a slight swelling at the ventral parts, representing the ventral lateral commissures, and the caudal end presents a cone shape and is frequently curved ventrally. The cephalic extremity can not be differentiated from the rest of the body and presents a spherical cap at the end. The caudal end of males also forms a long spiral shape with about 3.5 rounds. The esophagus of both males and females presents a glandular portion of about a third of its total length.

To date, adults of *Cercopithifilaria* sp. II have not been described, which is a major research gap that should be filled.

## Morphology of microfilariae

**Fig. 2:**
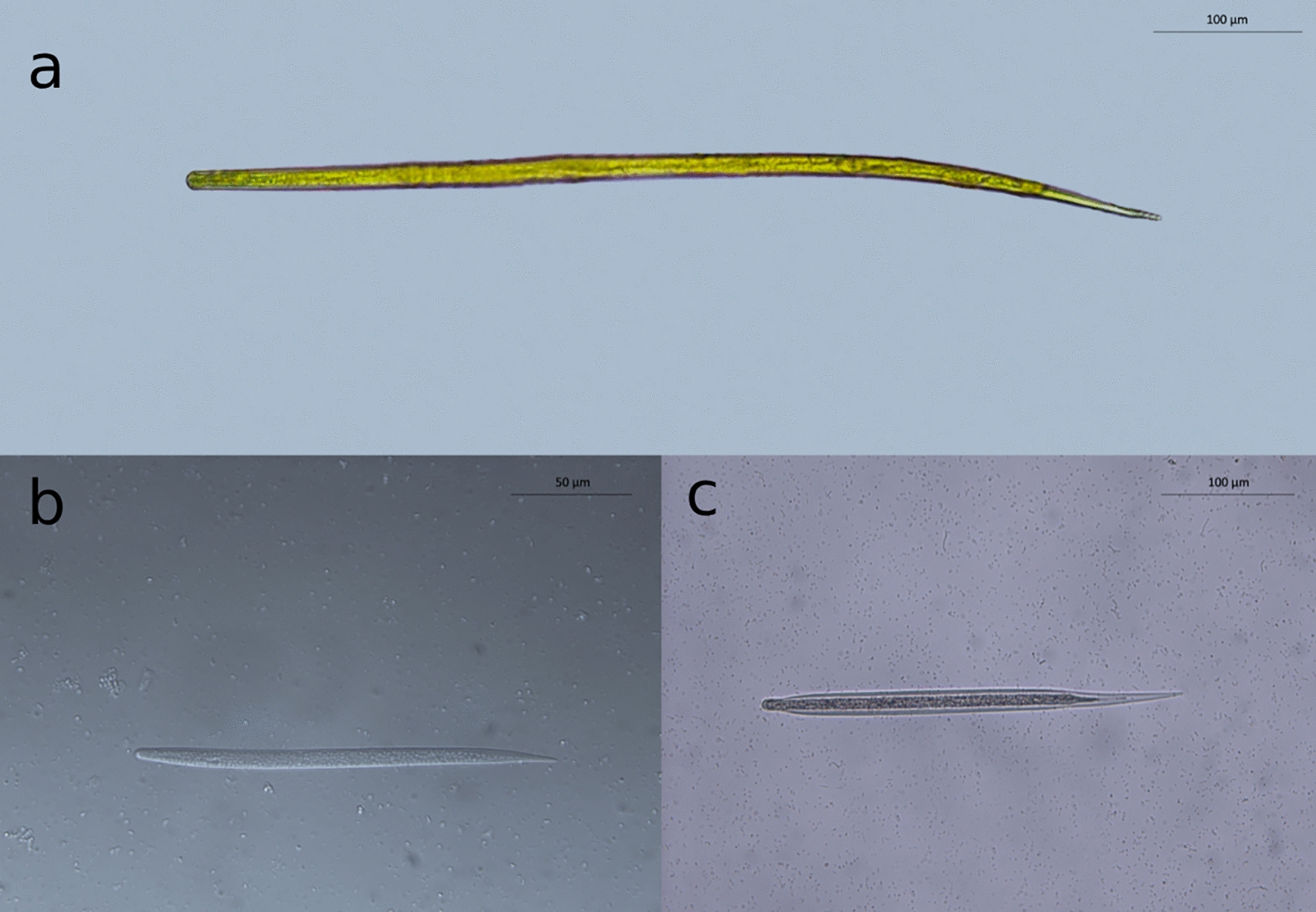
Microfilariae of *Cercopithifilaria* spp. **a** *Cercopithifilaria grassii*, **b** *Cercopithifilaria bainae*, **c** *Cercopithifilaria* sp. II

Microfilariae of *Cercopithifilaria* spp. are not found in the blood since they are skin-dwelling filarioids. The morphology of these immature stages is instrumental to the diagnosis of these filarioids in dogs, with the morphological features of mfs distinguishable through microscopical examination of the sediment from skin fragments soaked in physiological saline [7, 62]. In general, mfs of *C. bainae* are smaller than those of *C. grassii* and *Cercopithifilaria* sp. II (Fig. 2). The main morphological characters and morphometrical aspects on which the three species infecting dogs are differentiated are summarized in Table 3.

**Table 3:** Morphological and morphometrical parameters of microfilariae of *Cercopithifilaria* spp. of dogs

Morphometrical parameters	*Cercopithifilaria grassii* (Fig. 2a)	*Cercopithifilaria bainae* (Fig. 2b)	*Cercopithifilaria* sp. II (Fig. 2c)
Length (μm)	645–670	182–190	280–305
width (μm)	15–17	8.5–11	12–15
Cephalic end	Rounded with sclerotized convex formation and a tiny left hook at the top	Rounded with a small protuberance bearing a tiny cephalic hook	Rounded with a tiny left subterminal cephalic hook
Body	Cylindrical with thick cuticle and striae, which are interrupted in the lateral plane	Short and flattened dorso-ventrally, presenting a thick cuticle with transverse striations	Filiform with wide lateral alae
Tail	Long, thick and conical, with a blunt extremity that may appear bifid depending on the orientation	Pointed	Long, robust and conical

## Morphology of larval stages in tick vectors

The description of larval stages in the tick vector along with the molecular confirmation is of substantial importance to avoid misdiagnosis with other parasites. For example, Olmeda-García and Rodríguez-Rodríguez [63] reported *Acanthocheilonema dracunculoides* in *R. sanguineus* s.l. ticks that had fed on a positive dog, suggesting that this nematode could be vectored by this tick species. However, *A. dracunculoides* is known to be transmitted by hippoboscid flies of the species *Hippobosca longipennis* [64], which makes the former finding rather odd. In the study of Olmeda-García and Rodríguez-Rodríguez [63], molecular confirmation was not performed, and the dog, which was positive for circulating mfs of *A. dracunculoides* in the blood, was probably co-infected by *Cercopithifilaria* spp. Indeed, based on examination of the morphology of larval stages, the parasites reported by Olmeda-García and Rodríguez-Rodríguez [63] probably belong to the *Cercopithifilaria* genus. For example, the third-stage larvae (L3s) were much smaller (mean 2078.9 μm) than those of *A. dracunculoides* in the original description, in which larvae averaged 2400 μm [64]. In addition, characters such as morphology of the tail (i.e. two lateral conical lappets and one dorsal conical point) and esophagus (i.e. anterior muscular and longer posterior glandular portions) were compatible with the L3 of *C. bainae* described and molecularly confirmed in other studies [57].

*Rhipicephalus sanguineus* s.l. ticks may harbor different larval stages of *Cercopithifilaria* spp., including first-stage larvae (L1s), second-stage larvae (L2s) and L3s [14, 57]. Particularly for *C. bainae*, the morphology of the different larval stages present in the tick vector has been described in studies on the biological life cycle of this filarioid species [57]. The L1 presents a morphology similar to that of mfs, with a length of approximately 190 μm and width of approximately 5.5 μm, a rounded apical end, short tail and smooth transversal striated cuticle. The L2 presents a mean body length of approximately 798 μm and a width of approximately 26 μm and are characterized by the presence of the first molt exuvium at their anterior and posterior ends, a rounded apical end and a conical tail. The L3 presents a mean length of approximately 1700 μm and a width of approximately 27 μm. The buccal cavity is shallow, and the esophagus is divided into an anterior muscular and a longer posterior glandular region, respectively. The tail of L3s is slightly bent ventrally, presenting a rounded extremity with two lateral conical lappets and one dorsal conical point [57].

## Biological life cycle

The biology of *Cercopithifilaria* spp. has received more attention in the last decades following the detection of mfs of these skin-dwelling filarioids in dogs from several regions across the globe [15, 21–23, 28, 31]. The finding of infective L3s of *C. grassii* in *R. sanguineus* s.l. ticks from northern Italy [54] and Switzerland [52] in the early 1980s provided an incentive for investigating this tick species as an intermediate host for *Cercopithifilaria* spp. [57]. The role of brown dog ticks as intermediate hosts/vectors of *C. bainae* was assessed under experimental conditions, in which mfs ingested by nymphs that had fed on an infected dog were able to develop into L3s [57]. Concomitantly, larval stages of *Cercopithifilaria* spp. were detected in *R. sanguineus* s.l. ticks from Italy, Spain and Greece [14]. In later studies, L3s of *C. bainae* were also found in *R. sanguineus* s.l. ticks from Italy [24] and Brazil [32, 33] based on tick dissection and molecular identification.

**Fig. 3:**
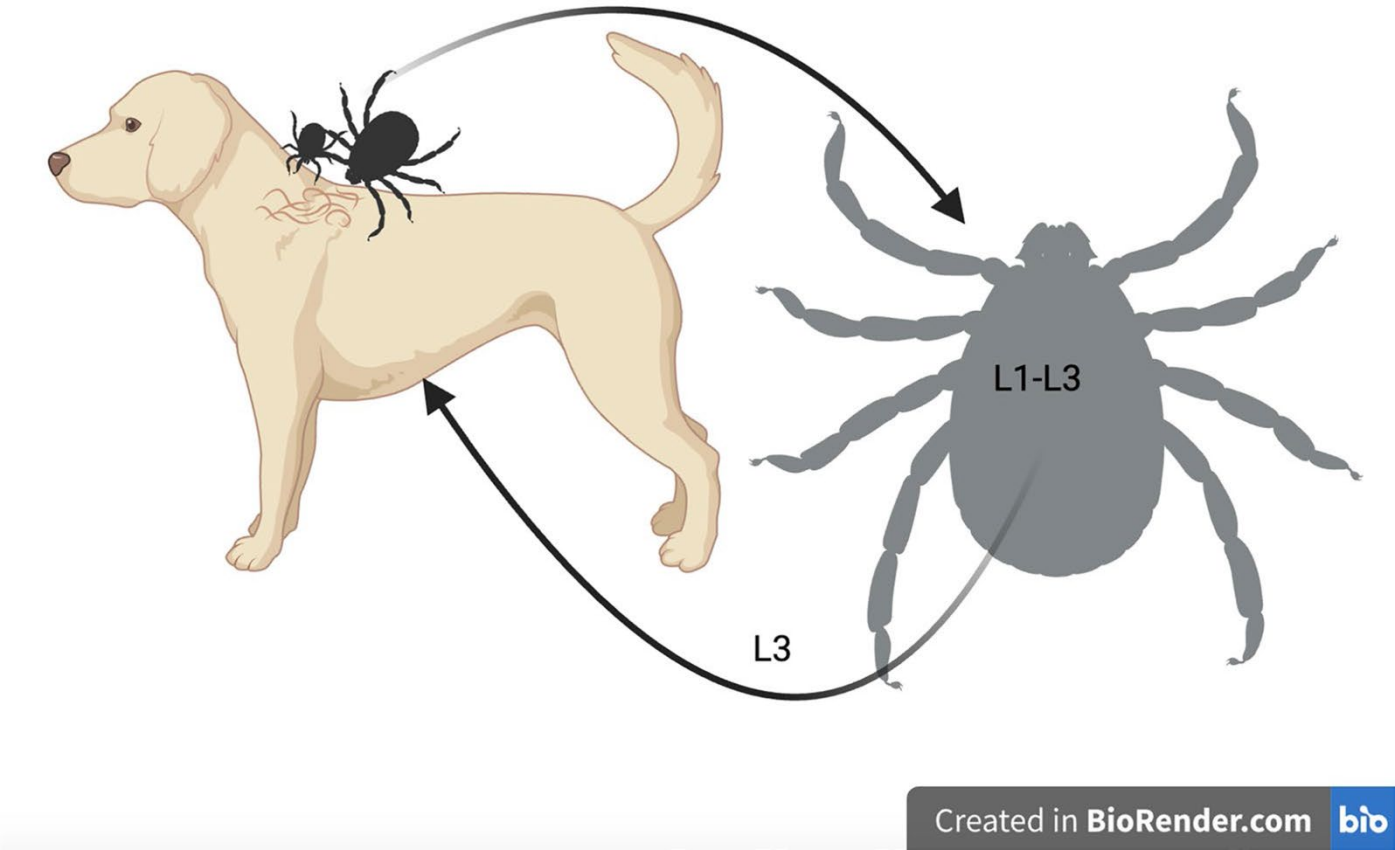
Biological life-cycle of *Cercopithifilaria bainae*. Microfilariae of the subcutaneous filarioid are distributed in the superficial dermal tissues of infested dogs. They are ingested by *Rhipicephalus sanguineus* sensu lato ticks at any life stage of the vector. Within the tick gut the microfilaria undergo further development into L1s, L2s and L3s (i.e. infective stages). The nematode reaches the infective stage (L3) within the tick in about 30 days after the blood meal of the tick. Finally, the L3s are transmitted to dogs by infected nymphs or adult ticks, and in the definitive host these larvae will undergo further development until they become adults, which are usually found beneath the subcutaneous tissues. L1, L2, L3, Larval stages 1, 2, 3, respectively

Although the life cycle of *Cercopithifilaria* spp. seems to be similar among the three species infecting dogs, most studies investigating the biology of these filarioids have been performed for *C. bainae* (Fig. 3). Mfs of *C. bainae* are distributed unevenly in the superficial dermal tissues of infested dogs, being mostly present on the skin of head, ears and neck regions [62], which are also among the most common attachment sites of *R. sanguineus* s.l. ticks [65]. The latter may acquire the infection during all life stages (i.e. larvae, nymphs and adults) by ingesting mfs, which subsequently develop into L1s (approx. 10 days), L2 (approx. 20 days) and L3s (approx. 30 days) within the tick gut [57]. Transstadial passage of the infection has been demonstrated in brown dog ticks, with infected nymphs becoming infected adults [57]; transovarial transmission is unlikely. Finally, L3s are transmitted to dogs by infected *R. sanguineus* s.l. nymphs or adult ticks, and in the definitive host these larvae will undergo further development until they become adults, which are usually located in the subcutaneous tissues [16, 57]. However, the period of development of larval stages to adults in dogs is unknown.

## Epidemiology

**Fig. 4:**
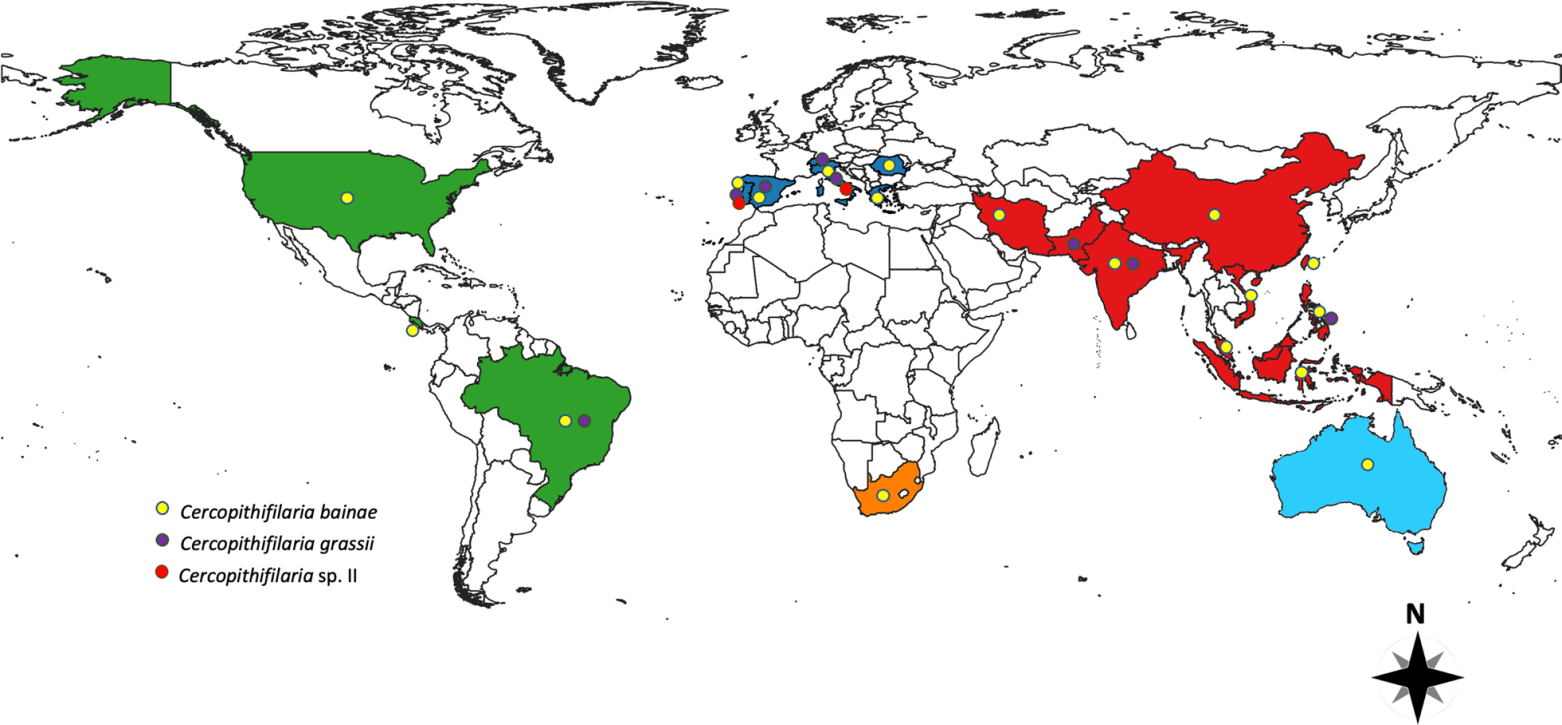
Biological life-cycle of *Cercopithifilaria bainae*. Microfilariae of the subcutaneous filarioid are distributed in the superficial dermal tissues of infested dogs. They are ingested by *Rhipicephalus sanguineus* sensu lato ticks at any life stage of the vector. Within the tick gut the microfilaria undergo further development into L1s, L2s and L3s (i.e. infective stages). The nematode reaches the infective stage (L3) within the tick in about 30 days after the blood meal of the tick. Finally, the L3s are transmitted to dogs by infected nymphs or adult ticks, and in the definitive host these larvae will undergo further development until they become adults, which are usually found beneath the subcutaneous tissues. L1, L2, L3, Larval stages 1, 2, 3, respectively

In the first multicenter study on the detection of *Cercopithifilaria* spp. in dog skin samples (*n* = 917) and *R. sanguineus* s.l. ticks (*n* = 890) from Italy, Spain and Greece, the overall prevalence in dogs was 13.9% based on microscopy of skin sediments and 10.5% based on molecular analysis [14]; these results indicate that these parasites were widespread in dogs from the Mediterranean basin. In this same study, a prevalence of *Cercopithifilaria* spp. in *R. sanguineus* s.l. ticks ranging from 5.2% to 16.7% at dissection was recorded [14]. In this study, the occurrence of these filarioid overlapped with the distribution of its tick vector, a finding that was further confirmed in other reports (see Fig. 4). The wide distribution of *C. bainae* in canine populations paralleled the high nucleotide variation among mitochondrial cytochrome *c* oxidase subunit 1 (*cox*1) sequences from skin samples and ticks, with up to 14 haplotypes characterized [66].

Studies on the occurrence of these nematodes in other tick species have been performed, but only *R. sanguineus* s.l. ticks have been demonstrated to act as competent vectors. For example, a study performed in Italy showed that *C. bainae* were unable to develop in *I. ricinus* ticks collected on a positive dog but that they fully developed into L3 in *R. sanguineus* s.l. that fed on the same animal concomitantly [67]. Furthermore, *C. bainae* has been molecularly detected in *Dermacentor reticulatus* ticks in Romania [68] and in *Rhipicephalus haemaphysaloides* in India [20]; however, the development of this filarioid in these tick species up to L3 needs to be confirmed.

## Pathology

*Cercopithifilaria* spp. usually cause sub-clinical infections and was considered for a long time to be non-pathogenic [14]. However, with the increase in the number of studies on these parasites in dogs, an increasing number of clinical signs, such as dermatitis characterized by erythema, papule, pruritus, non-healing and ulcerative skin lesions and subcutaneous nodules, have been described [28, 62]. In addition, several mfs of *C. bainae* have been found in a giant cutaneous cyst (diameter 15 cm) in a dog from Brazil [69]. Non-dermatological signs have also been described, including a case of chronic polyarthritis in a *C. bainae*-infected dog from Italy [70]. Numerous mfs were observed in the synovial fluids collected from the joints of this dog and other common causes of chronic polyarthritis were ruled out [70]. Apart from these direct clinical presentations, the role of *Cercopithifilaria* spp. in facilitating infection by other tick-borne pathogens has also been proposed; however, no specific studies have been performed to validate this hypothesis [25]. While the actual pathogenic role of *Cercopithifilaria* spp. is not yet completely elucidated, the awareness of veterinary practitioners regarding these subcutaneous filarioids in the differential diagnosis of skin diseases of dogs should be increased.

## Diagnosis and treatment

**Fig. 5:**
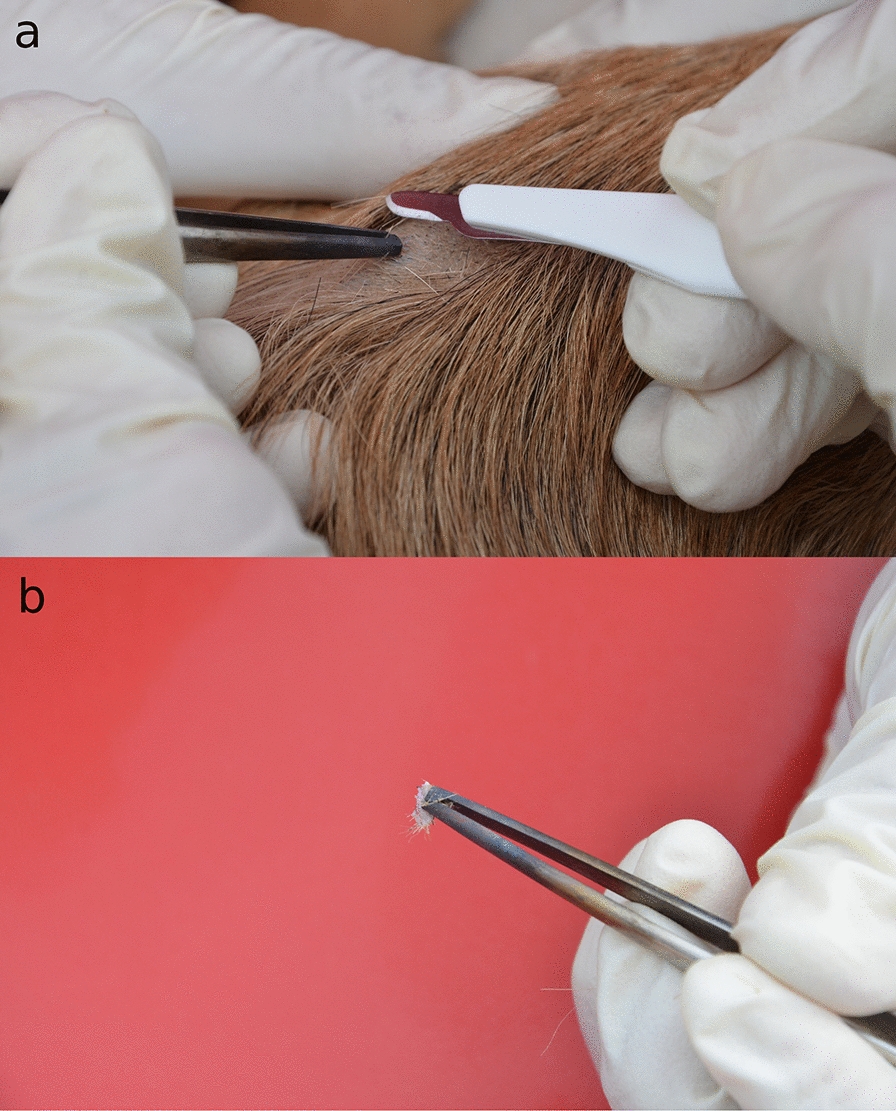
Skin biopsy procedure in a dog for the detection of dermal filarioids. **a** Anatomical site on the dog (inter-scapular region). **b** Skin fragment collected

**Fig. 6:**
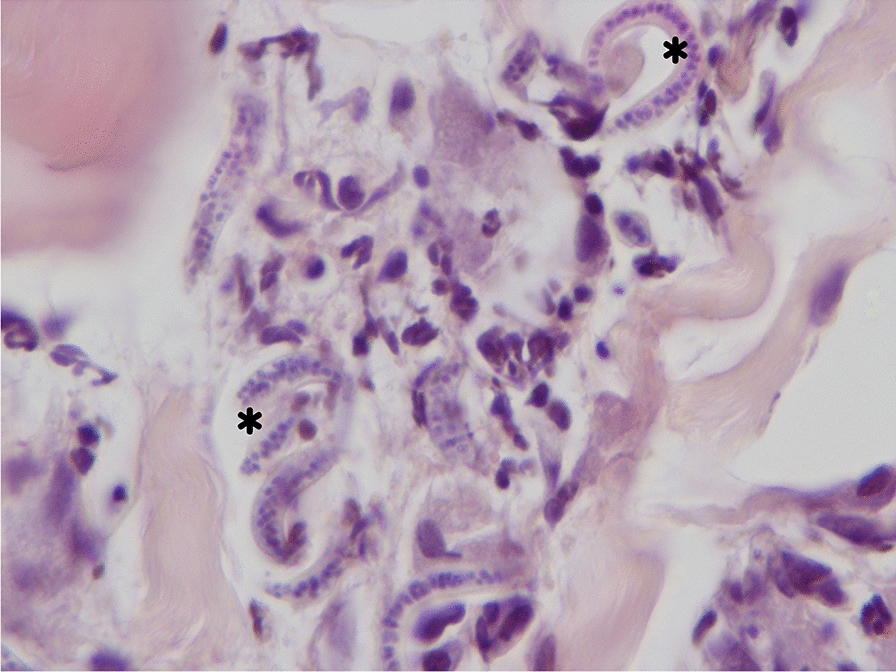
Histopathology from skin. In the interstitium of the dermis are neutrophils, a few eosinophils and microfilariae of *Cercopithifilaria* sp. (asterisk). Hematoxylin–eosin stain. Images are courtesy of Francesca Abramo

The diagnosis of *Cercopithifilaria* spp. requires an invasive and challenging procedure, which is not always accepted by pet owners and, therefore, it is not standardly performed by veterinary practitioners [14, 15]. The procedure consists of an initial step involving disinfection of the skin area to be sampled with 70% alcohol and 4% chlorhexidine solution followed by application of a local anesthesia with 2% lidocaine hydrochloride. This is followed by the collection of a deep skin sample fragment from the shoulder or inter-scapular region using a scalpel or a biopsy punch (Fig. 5). The skin sample is then soaked in 2 ml of saline solution (NaCl 0.9%) overnight at room temperature, following which the sediment is observed under the light microscope for the presence of mfs [14, 15]. Part of the skin sample and the remaining sediment should be frozen at − 20 °C for molecular analyses, which is usually performed with the use of primers that amplify a portion of the *cox*1 gene [71]. Histopathological examination may also be performed for the observation of mfs in skin lesions (Fig. 6).

Studies describing the treatment of *Cercopithifilaria* spp. in dogs are scant. To date, only one field study performed on privately owned dogs from Portugal has demonstrated that a single treatment with a spot-on formulation containing imidacloprid 10% and moxidectin 2.5% is efficacious in eliminating mfs of *Cercopithifilaria* spp. from dogs [15]; a dog presenting erythematous lesions and affected by *C. bainae* was also successfully treated with the same formulation in the USA [28]. The above studies support the notion that macrocyclic lactones are efficacious for the treatment of skin-dwelling mfs of *Cercopithifilaria* spp. However, the effect of these drugs may be influenced by the aberrant location of mfs. For example, the treatment with milbemycin oxime, administered orally at the dose of 0.5 mg/kg, once per week for 3 consecutive weeks did not eliminate mfs from the synovial fluid of a dog presenting chronic polyarthritis [70]. Similarly, a single treatment with a spot-on formulation containing imidacloprid 10% and moxidectin 2.5% did not eliminate mfs of *C. bainae* from the fluid of a giant cyst of a dog, which was only resolved after the surgical removal of the cyst and treatment with oral ivermectin at a dose of 0.3 mg/kg, daily for 7 days, and of 0.6 mg/kg, daily, for 7 additional days [69], even though the persistence of mfs was not continuously evaluated. For the latter treatment, the dose of ivermectin would be lethal for dogs with the multidrug resistance mutation 1 (MDR1) gene defect, therefore, animals should be tested for this gene defect prior to treatment [72].

## Conclusion

In this article, information on *Cercopithifilaria* spp. has been assessed to provide an overview of these little known but widespread parasites of potential clinical significance. Indeed, it would appear that differently from what was previously thought, these filarioids may cause clinical disease in dogs, which is mainly associated with dermatological disorders, but may also be associated with other clinical presentations, such as chronic polyarthritis, possibly in the formation of cutaneous cysts. Recent studies have demonstrated that these parasites have a wide distribution, overlapping with the occurrence of their tick vector (*R. sanguineus* s.l.). Therefore, veterinary practitioners should be aware of *Cercopithifilaria* spp. and include these filarioids in the differential diagnosis of skin diseases of dogs.

## Data Availability

Not applicable.

## Abbreviations

MDR1: Multidrug resistance mutation
Mfs: Microfilariae
